# Supplementary material for: Comprehensive Analysis of Virulence Determinants and Genomic Islands of blaNDM-1-Producing Enterobacter hormaechei Clinical Isolates from Greece
Source: Antibiotics (Basel). 2023 Oct 18;12(10):1549. doi: 10.3390/antibiotics12101549 (PMC10604629; doi:10.3390/antibiotics12101549)
Supplement: Supplementary file 1 [file antibiotics-12-01549-s001.zip › antibiotics-2654594-supplementary-revised-1.pdf]

Table S1. Antimicrobial susceptibility testing of strains EC-ML559 and EC-ML621.

**Antimicrobial agent**	**MIC (mg/L)**	
	**EC-ML559**	**EC-ML621**
Cefotaxime	≥ 64 (R)	≥ 64 (R)
Ceftazidime	≥ 64 (R)	≥ 64 (R)
Ceftriaxone	≥ 64 (R)	≥ 64 (R)
Cefepime	≥ 64 (R)	≥ 64 (R)
Ertapenem	≥ 8 (R)	≥ 8 (R)
Imipenem	≥ 16 (R)	8 (R)
Meropenem	≥ 16 (R)	≥ 16 (R)
Aztreonam	2 (R)	≥ 64 (R)
Amoxicillin/Clavulanic acid	≥ 32 (R)	≥ 32 (R)
Ticarcillin/Clavulanic acid	≥ 128 (R)	≥ 128 (R)
Piperacillin/Tazobactam	≥ 128 (R)	≥ 128 (R)
Ciprofloxacin	≥ 4 (R)	≥ 4 (R)
Ofloxacin	≥ 8 (R)	≥ 8 (R)
Tetracycline	≥ 16 (R)	≥ 16 (R)
Trimethoprim/Sulfamethoxazole	≥ 320 (R)	≥ 320 (R)
Amikacin	16 (S)	≥ 64 (R)
Gentamicin	≤ 1 (S)	≥ 16 (R)
Tobramycin	≥ 16 (R)	≥ 16 (R)
Colistin	≤ 0.5 (S)	≤ 0.5 (S)
Tigecycline	2 (S)	1 (S)

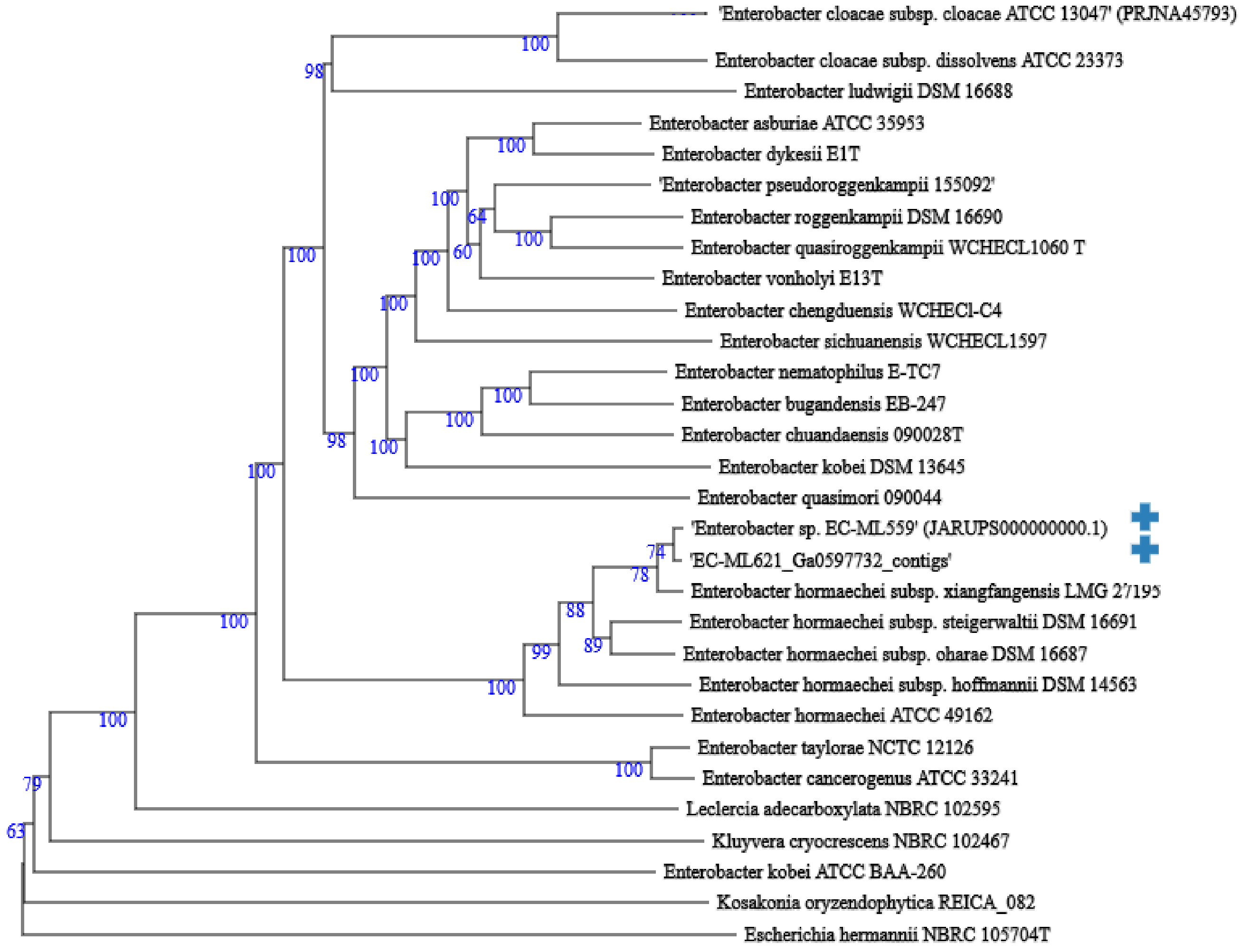

Figure S1. Tree inferred with FastME 2.1.6.1. from GBDP distances calculated from genome sequences. The branch lengths are scaled in terms of GBDP distance formula *d5*. The numbers above branches are GBDP pseudo-bootstrap support values > 60% from 100 replications, with an average branch support of 92.5%. The tree was rooted at the midpoint.

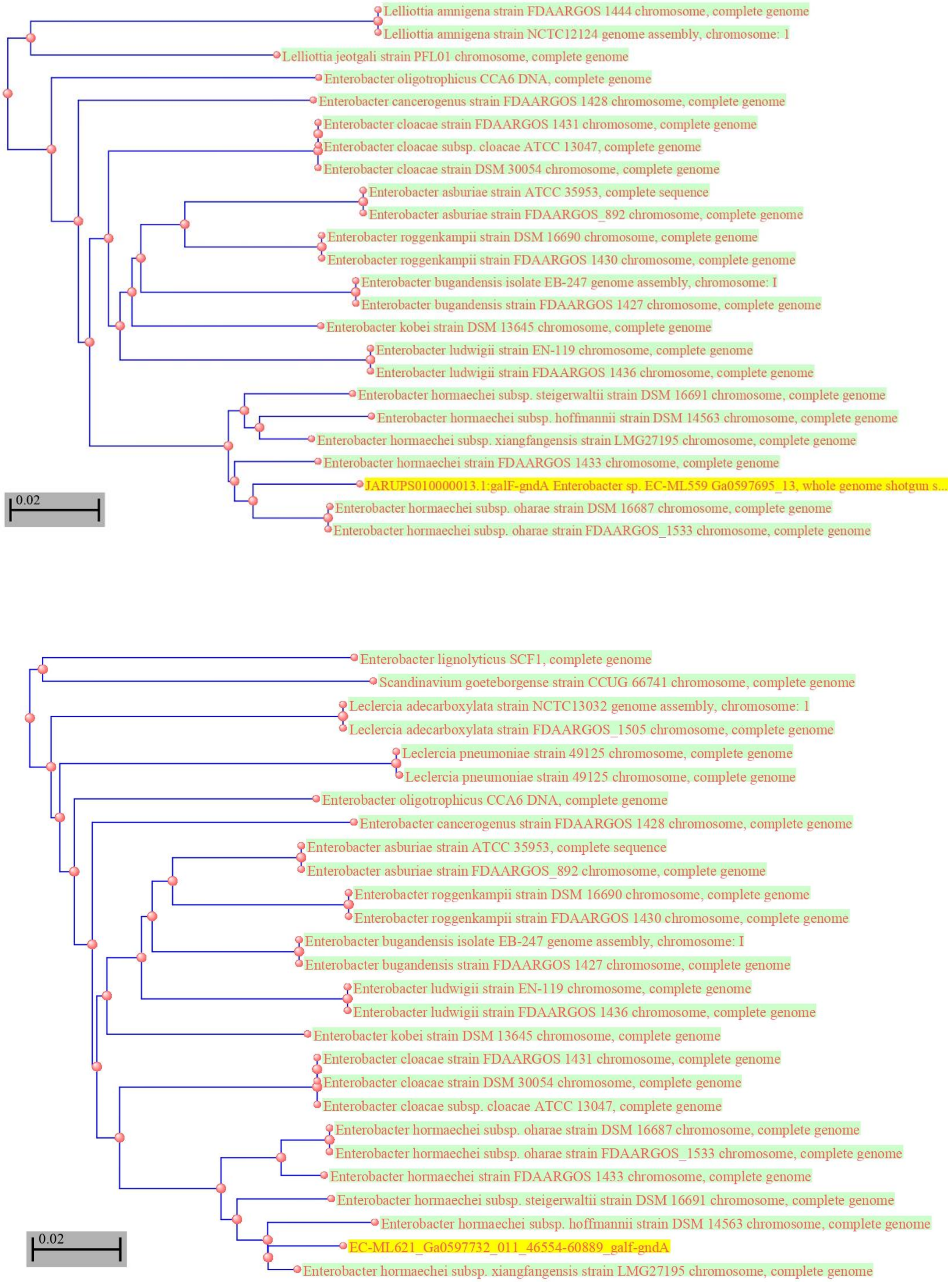

Figure S2. Phylogenetic analysis of the O-antigen clusters of strains EC-ML559 and EC-ML621 compared with other *Enterobacter cloacae* complex type strains.

EC-ML559

Aligned against reference genome *Enterobacter xiangfangensis* strain LMG27195, complete genome

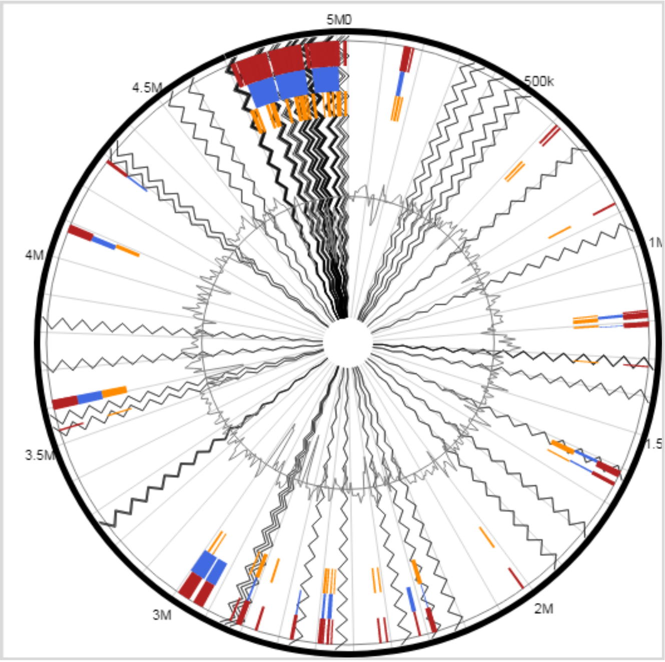

EC-ML621

Aligned against reference genome *Enterobacter xiangfangensis* strain LMG27195, complete genome

Legend

Prediction Methods

- Integrated
- IslandPath-DIMOB
- SIGI-HMM
- IslandPick (No results found - unavailable for draft genomes)
- Islander (Not available)

Contigs & Alignment

- Contig Boundary
- Alignment

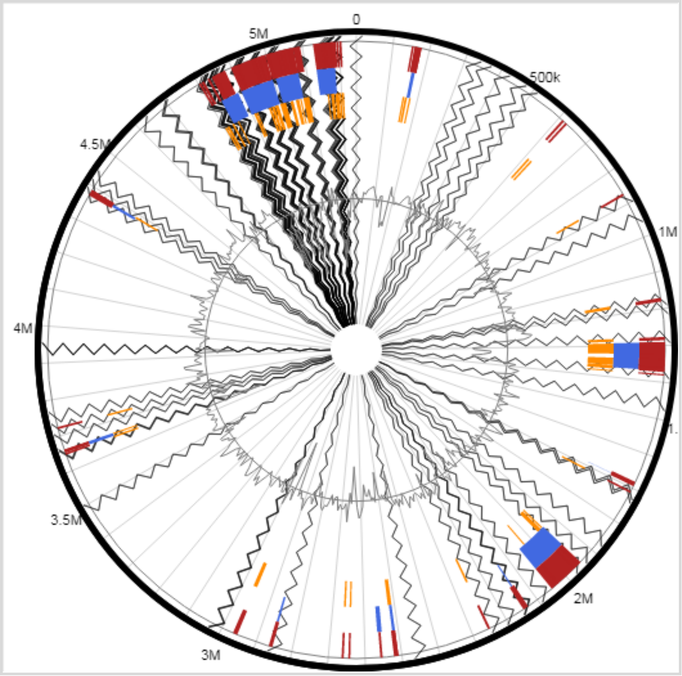

Figure S3. Predicted genomic islands and virulence/resistance gene annotations (when available) are colored within the images based on the legend. Gaps are indicated as grey bars in the circular plot, and any unaligned contigs are displayed at the end of the genome plot (indicated in grey on the circular alignment plot).

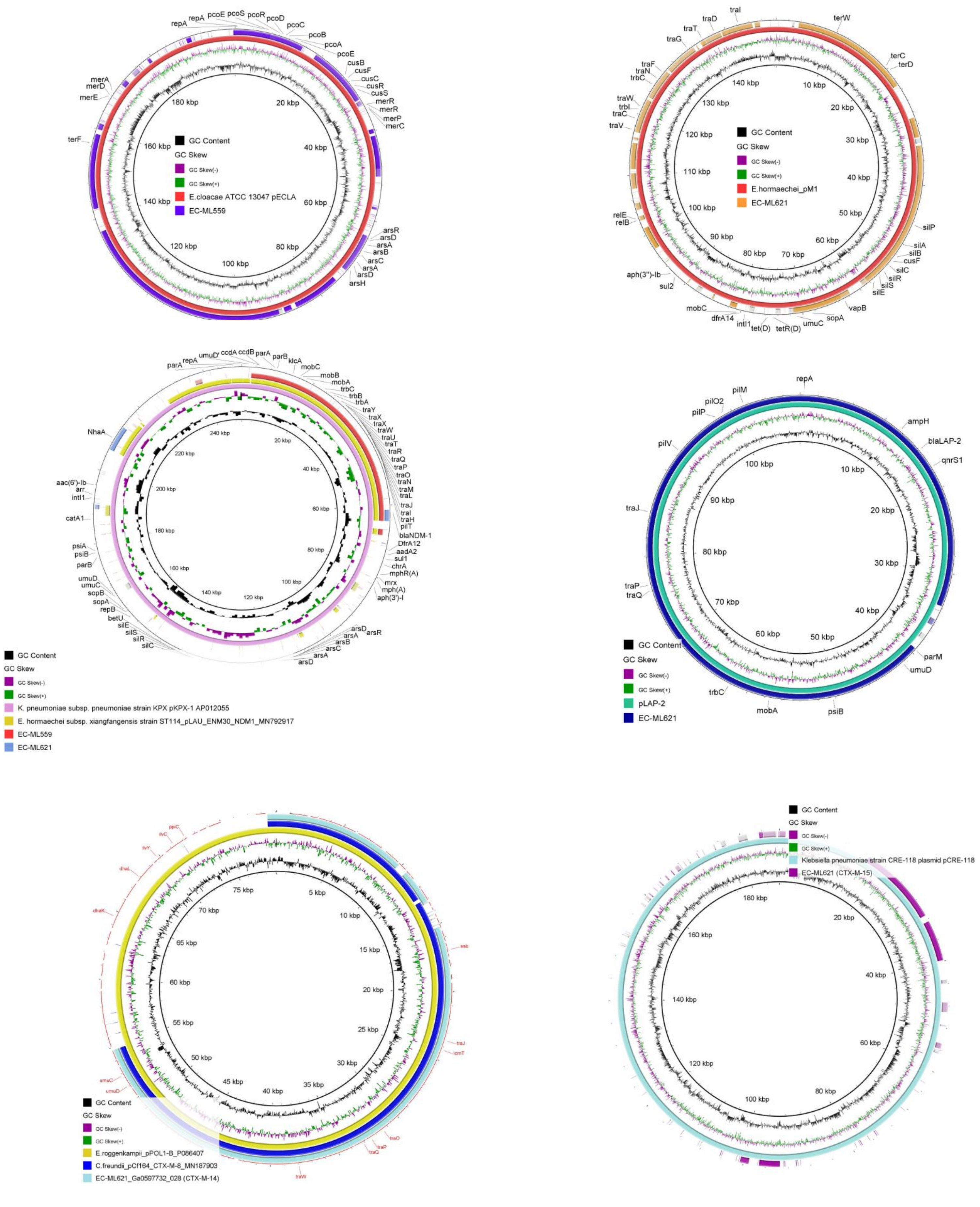

Figure S4. BlastN comparisons of β-lactamase harboring sequences of strains EC-ML559 and EC-ML621 identified *in silico* with those published plasmid sequences.

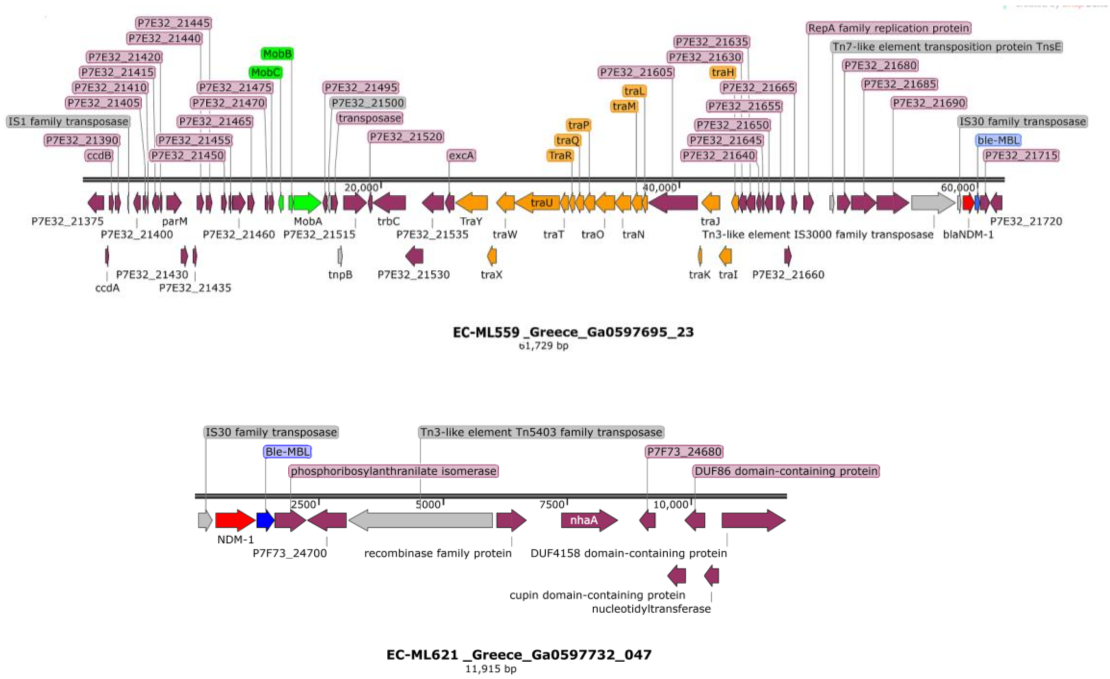

Figure S5. Genetic background of *bla*NDM-1-harbouring contigs of strains EC-ML559 and EC-ML621.
